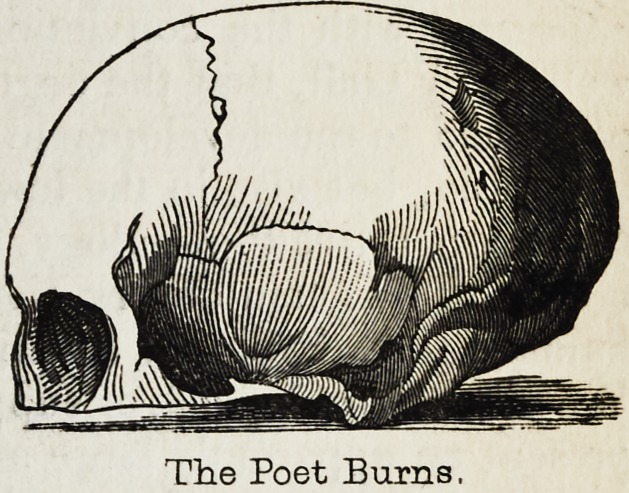# On the Origin of the Moral Qualities and Intellectual Faculties of Man, &c.

**Published:** 1840-01

**Authors:** 


### ART. VII.

1. *On the Origin of the Moral Qualities and Intellectual Faculties of Man, and the Conditions of their Manifestation.* By FRANÇOIS JOSEPH GALL, M.D. Translated from the French, by WINSLOW LEWIS, Jun., M.D.—*Boston, U.S.*, 1835. Six vols. 12mo.

2. *Phrenology, or the Doctrine of the Mental Phenomena.* By J. G. SPURZHEIM, M.D. *Fourth American Edition.—Boston, U.S.*, 1835. Two vols. 8vo.

3. *Traité de Phrénologie Humaine et Comparée; accompagné d'un magnifique Atlas, in folio, de* 120 *Planches, contenant plus de* 600 *sujets d'Anatomie Humaine et Comparée.* Par J. VIMONT, M.D., &c. —*Paris et Londres*, 1835. Two vols. 4to.

4. *Cours de Phrénologie.* Par F. J. V. BROUSSAIS, Membre de l'Institut, &c.—*Paris et Londres*, 1836. 8vo.

*Lectures on Phrenology.* By F. J. V. BROUSSAIS, M.D., &c.—*Paris*, 1836.

5. *A System of Phrenology.* By GEORGE COMBE. Fourth Edition.—*Edinburgh*, 1837. Two vols. 8vo.

6. *Elements of Phrenology.* By GEORGE COMBE. Fourth Edition.—*Edinburgh*, 1837. 12mo.

7. *The Constitution of Man considered in Relation to External Objects.* By GEORGE COMBE.—*Edinburgh*, 1837 and 1839. 12mo, Seventh Edition; and Royal 8vo, Seventh Impression.

8. *On the Functions of the Cerebellum.* By Drs. GALL, VIMONT, and BROUSSAIS. Translated from the French by GEORGE COMBE. *Also Answers to the Objections urged against Phrenology by Drs. Roget, Rudolphi, Prichard, and Tiedemann.* By GEORGE COMBE and ANDREW COMBE, M.D.—*Edinburgh*, 1838. 8vo.

9. *The Phrenological Journal.* Nos. I.—LXI.—*Edinburgh and London*, 1823-39. 8vo.

10. *Selections from the Phrenological Journal; comprising Forty Articles in the First Five Volumes, chiefly by George Combe, James Simpson, and Dr. Andrew Combe.* Edited by ROBERT COX. —*Edinburgh*, 1836. 12mo.

11. *Statistics of Phrenology: being a Sketch of the Progress and Present State of that Science in the British Islands.* By HEWETT C. WATSON. —*London*, 1836. 12mo.

12. *An Introduction to Phrenology.* By ROBERT MACKNISH, LL.D. *Second Edition.—Glasgow*, 1837. 12mo.

13. *The Principles of Phrenology.* By SIDNEY SMITH.—*Edinburgh*, 1838. 8vo.

14. *A Treatise on Insanity and other Disorders affecting the Mind. (Supplementary Note on Peculiar Configurations of the Skull, with Observations on the Evidence of Phrenology.)—London*, 1835. 8vo.

15. *Phrenology Vindicated, and Anti-Phrenology Unmasked.* By CHARLES CALDWELL, M.D.—*New York*, 1838. 12mo.

16. *Observations on Mental Derangement: being an Application of the Principles of Phrenology to the Elucidation of the Causes, Symptoms, Nature, and Treatment of Insanity.* By ANDREW COMBE, M.D. —*Edinburgh*, 1830. Post 8vo.

17. *Compendio di Anatomia Fisiologico-Comparata, &c.* Del Dottore FILIPPO UCCELLI, Professore di Anatomia Umana e Comparata nell' Universita di Pisa, &c. &c.—*Firenze*, 1825. Six vols. 8vo.

*Compendium of Physiological and Comparative Anatomy, &c.* By Dr. PHILIPUCCELLI, Professor of Human and Comparative Anatomy in the University of Pisa, &c.—*Florence*, 1825. Six vols. 8vo. (Vol. iv. being an Exposition of Phrenology.)

18. *Lettera al Signor Defendente Sacchi, sul Merito e Valore della Craniologia, &c.*—*Milan*, 1836. 8vo.

*Letter to Signor Defendente Sacchi, on the Merits and Truth of Craniology.*—*Milan*, 1836.

19. *Memorie risguardanti la Dottrina Frenologica ed altre Scienze che con essa hauno stretto rapporto.* Da LUIGI FERRARESE, D.M., Medico Ordinario della Reale Casa dei Folli in Aversa, &c. &c.—*Napoli*, 1839. 8vo, pp. 98.

*Memoirs on the Phrenological Doctrines and other relative sciences.* By LUIGI FERRARESE, Physician in Ordinary to the Royal Asylum for the Insane at Aversa, &c. &c.—*Naples*, 1839.

20. *Caractères Phrènologiques et Physiognomoniques des Contemporains les plus célèbres selon les systêmes de Gall, Spurzheim, &c. Avec* 37 *Portraits.* Par THEODORE POUPIN.—*Paris*, 1837. 8vo. pp. 500.

*The Phrenological and Physiological Character of the most celebrated Men of the present day, according to the systems of Gall, Spurzheim, &c. With* 37 *Portraits.* By THEODORE POUPIN.—*Paris*, 1837.

21. *Two Treatises on Physiology and Phrenology* (*republished from the Seventh Edition of the Encyclopædia Britannica*). By P. M. ROGET, M.D.—*Edinburgh*, 1838. Two vols. 8vo.

22. *On the Physiology of the Brain as the Organ of the Mind.* (*Transactions of the Provincial Medical Association.* Vol. vii.) By CHARLES COWAN, M.D.—*Worcester*, 1839. 8vo.

23. *Medical Notes and Reflections.* By HENRY HOLLAND, M.D. (Chap. xxx. on Phrenology.)—*London*, 1839. 8vo.

THE time seems to us to have now arrived when a careful and conscientious examination of the truth and merits of phrenology has become imperative on every intelligent member of the profession, and when its claims to attention can no longer be safely neglected, even by those who are more concerned about their personal reputation than about the advancement of science and the improvement of mankind. If phrenology be true, its importance to medicine and to philosophy can scarcely be overrated, and no one can be more usefully employed than in advocating its cause; whereas, if it be false, and the observations on which it professes to rest be really incorrect, a great service would be rendered to medicine by at once demonstrating their hollowness, and directing the able and zealous exertions of its misled followers into a safer and more profitable channel. Acting on this conviction, we have ourselves lately bestowed much attention on the subject; and we feel that no apology can be required for now laying the results before our readers.

In contemplating the past history of phrenology, the difference of tone and manner in which it is now spoken of cannot fail to be remarked. Five and twenty years ago, when the late Dr. Gordon made his unprovoked and ungenerous attack in the Edinburgh Review on "the man of skulls," whom he imagined to have been slain in the same Review twelve years before by the abler hand of the late Dr. Thomas Brown,—the public, then profoundly ignorant of the merits of the question, went so heartily along with him in the torrent of invective, abuse, and ridicule, in which he so inconsiderately indulged, that for years after, the subject was never alluded to without a smile of contempt or a laugh of derision, and the gentlest fate which was assigned for it was that of speedy and eternal oblivion.

How different the state of things is now, few even of its most inveterate opponents require to be told. For years phrenology has ceased to be the subject of drawing-room gossip, or the favorite topic of the ridicule of the shallow. In mixed society it is as little heard of as any other branch of physiological or scientific enquiry, which the rules of good breeding naturally warn us to reserve for a more fitting occasion; and from this circumstance many imagine that it has wholly disappeared. But when we examine a little more closely what is passing around us, the signs of its vitality and growth are found so numerous and palpable as to shadow forth rather a long, and vigorous, and useful existence, than the speedy extinction with which it has been threatened. In proof of this, we would refer, among other things, to the numerous works which have lately appeared, not in this country only, but in America and on the continent, and the titles of some of which are prefixed to the present article, not for review, for that were impossible, but as indications of what is going on. We would refer, also, to the variety of quarters in which phrenology is already received, and more or less acted upon, as established truth. We confess, indeed, that, although far from inattentive to its later progress, we were not prepared for the numerous evidences of its extended diffusion which forced themselves upon our notice, without enquiry, in a late tour through part of England, Scotland, and the north of France, Paris included. In asylums, schools, and factories, we found it recognized and acted upon, where ten years before not a trace of its existence was to be heard of. Not only, however, are works on phrenology rapidly multiplying in number, but they are improving in character; and in accuracy of observation, sobriety of inference, and vigour of thinking, a few of them may bear a comparison with any physiological or philosophical works which have lately appeared. That these qualities have not been without their natural effect in exciting a widely diffused interest in the public mind, is evident from the extraordinary and steady sale which several of the phrenological works, the best, we believe, of their class, have met with, in the face of the active and influential hostility of the leading journals of the day, led on by Lord Jeffrey himself, in the Edinburgh Review, and also by the Quarterly. If this demand had lasted only for a year or two, it might have been plausibly enough ascribed to fashion and a love of novelty; but when it has extended, as in the instance of Mr. Combe's books, over a period of twenty years, it is difficult to account for it, except on the supposition of their possessing a real and abiding interest, derived either from the inherent nature of the subject, or from the manner in which it is treated. Not to mention the wide diffusion of the works of the founder of phrenology, and his colleague, Spurzheim, we have now before us the *sixty-first* quarterly number of the Phrenological Journal, which has been carried on for upwards of sixteen years, and, as we are told by the editor, is yearly increasing in circulation. We have also before us an advertisement of the last edition of Combe's "Constitution of Man considered in Relation to external Objects," in which it is mentioned that that work, being an application of phrenology to human improvement, continues in constant demand, after a sale of *forty-five thousand copies* in Great Britain and Ireland alone, besides large editions in America, and translations into French and German. The "System of Phrenology" of the same author, which contains the best exposition of the doctrine, its evidences and applications, although selling at a guinea, and therefore not likely to be bought without due consideration, has already gone through four editions, and, as we have learned, still continues in increasing demand, to the extent of 600 copies a year. In like manner, the "Introduction to Phrenology," by the late Dr. Macnish, have sold, as appears from the advertisement, to the very large extent of 5000 or 6000 copies within three years, notwithstanding the increasing number of competitors in the market. We might mention many other evidences, of a similar nature, to prove the progress which phrenology is making in public opinion; but for these we must refer the reader to the curious volume of Mr. Hewett Watson, on "The Statistics of Phrenology," in which an account is given of the various works published, and societies existing, in this country, and in which the reader will find much useful information, of an authentic kind, relating to the past history and present state of phrenology.

As further evidence, of a very unequivocal kind, we may refer to the numerous courses of lectures given on the subject within the last five years in most of our larger towns, and to the intelligent audiences by which they were attended. Even the frequent display of phrenological busts in the windows of shops is a sign not without meaning to reflecting minds. But perhaps more than all, the rapid diffusion of phrenological ideas under the cover of ordinary language, and without any reference to their true source, is a proof not only that the new philosophy is making progress, but that it is found to be of direct utility in questions of nervous disorder, insanity, education, morals, and crime. We are acquainted with medical and educational works which have gained no small repute, from the copious but unacknowledged use they have made of the doctrines of phrenology, and the reputation of which depends chiefly on their borrowed views. We have sometimes, indeed, been tempted to smile at the ready acceptance which strictly phrenological ideas have met with when thus stolen and offered at second-hand, only a little altered in dress to prevent their paternity being traced. But much as we rejoice in the diffusion of useful truth, we cannot refrain from condemning this plan of acquiring a temporary popularity at the expense of science; and we are glad that the risk of detection will soon become so great as to deter most men from such unscrupulous conduct. It may seem at first view a light matter thus to put forth a truth in disguise; but in reality, its forced separation from the principle which alone renders its application safe and advantageous, deprives it of much of its practical value; and it is for this reason, as well as for its dishonesty, that we object to the practice.

If our space permitted, we might further refer to the account given in the last number of the Phrenological Journal of Mr. Combe's progress in the United States, and to the works of Vimont, Broussais, Ferrarese, and other continental authors, to show that, abroad as well as at home, phrenology is exciting the serious attention of men of science. But we must content ourselves with the simple statement that such is the fact; and that, among the more recent of the French medical works, the principles of phrenology are either expressly or tacitly assumed, as if no doubt had ever been entertained regarding them. Many hesitate, and justly, about the details, but we do not go too far in affirming that a conviction of the truth of the leading principles of the new physiology of the brain is fast diffusing itself over the continent.

With these facts before us, we need scarcely add that our past silence has not arisen either from participating in the contempt with which phrenology was formerly treated, or from having been unobservant of its more recent progress. From the first we saw that, whether true or false, the subject was one of great extent and serious import; and we delayed forming or expressing any opinion till we should have sufficient time and opportunity to verify its principles and scrutinize its details. Having now done so, sufficiently to qualify ourselves for giving an opinion, we should shrink from our duty, both to our readers and to science, were we to hesitate longer in avowing our conviction that phrenology embodies many facts and views of great general interest, and direct practical utility to the physician, the philosopher, and the philanthropist; and that as such, it has established a claim to a more careful, serious, and impartial examination on the part of the profession than it has ever yet received. We do not by this mean to affirm that all the facts and doctrines taught by the phrenologists are accurate and true; so far from it, we have satisfied ourselves that many have been admitted without a sufficiently scrupulous examination; and that not seldom, the conclusions deduced from them have been pushed beyond the limits of strictly logical inference. We are consequently not inclined to adopt either of them without due verification. But it would be the height of injustice were we on that account to reject the whole as unfounded, and to maintain that they cannot possibly be true, merely because they are in contradiction to our own preconceived opinions; and yet, to the most unphilosophical and illogical mode of proceeding we have condemned, may be traced almost all the opposition which Gall's discovery has met with.

If the functions of the brain had been already ascertained by some method of enquiry of a more satisfactory nature than that resorted to by Dr. Gall, we might have argued, with some fairness, that if his observations were inconsistent with those already obtained, they could not possibly be true. But when it is notorious that all other methods of investigation *have failed* to unfold the mystery of the cerebral functions, it is as obvious as the noonday sun, that no information which we may possess can enable us to decide *a priori*, and *without any examination of the evidence*, that his mode of enquiry is fallacious and its results untrue. To entitle the judgment of any one to the least weight, either for or against the reality of the discovery, it must be based upon a careful examination of the facts and evidence. If a man propounds as a new discovery that the function of the liver is to secrete milk, we are logically entitled to disregard his assertion, because we are already in possession of demonstrative evidence that the function of the liver is to secrete bile. But it is very different with the case of the brain. When Dr. Gall affirms, that by a new mode of enquiry, easy of practice, he has ascertained that the anterior lobes of the brain serve for the manifestation of intellect, the posterior lobes for that of the animal passions, and the coronal region for that of the moral feelings, we have no right whatever, either in sense or in philosophy, to say, "No! this is a mistake." So long as we do not possess a shadow of information at variance with his assertion, it would be to assume in profound ignorance the privilege of Omniscience to say, that such a thing "*cannot be.*" With regard to the brain, we are in precisely the same situation as we would be with regard to the spleen, if some physiologist were to discover that its use was to secrete a particular kind of digestive fluid, and were to describe how he made the discovery, and how it might be verified. If the greatest philosopher that ever lived were thereupon to deny, *without examination of the evidence*, that the spleen served for any such purpose, who would attach any weight to his objection, or who would care one straw for the adverse opinion of any man who had not thought it worth his while *to test the fact*, before deciding upon its truth? In like manner, when Gall professes to have found out the functions of the brain, and explains how he made the discovery and how it may be verified, it would be equally childish and futile to satisfy ourselves with the simple denial without direct examination of the fact, that the different organs above specified serve the purposes pointed out by him. Either we must meet the question of fact by a personal and extensive appeal to nature, or we ought to avow that we are not prepared to speak definitely as to the truth of the doctrine.

We are aware that many talk of phrenology as a mere theory, invented by the fertile imagination of an enthusiast, and under this impression think they treat it with all due respect, when they give it half an hour's consideration before they express an opinion of its merits. We confess that we ourselves once belonged to this rather numerous class of persons, and that we extracted much amusement from the pages of Gall and Spurzheim, by a playful travestie of some of the curious anecdotes by which they occasionally illustrate their positions; and which, considered apart from the context, have often a somewhat ludicrous aspect. But when at length we came into contact with Spurzheim himself, and remarked, instead of the wild enthusiasm of a visionary, the truthful earnestness, the calm and forcible appeals to fact and reason, and the occasionally almost solemn feeling of the importance of his mission, with which he advocated his cause, we felt that the subject was of too grave a nature to be either hastily admitted or slightingly rejected, and resolved to try his positions by the strict test of observation before finally deciding upon their truth. The result was, as we have already said, not the blind adoption of the whole phrenological doctrines, but a growing and conscientious conviction of the soundness of the great principles on which they are based, and of the practical value of many of their details. But although we see strong grounds for believing that an imperishable foundation has been laid, the edifice itself is still far from being complete, and many years and much labour will be required to bring it to that perfection of which even its present outline shows it to be susceptible, and which, in their short-sightedness, some of its admirers imagine it already to have attained.

Gall's discovery, if such it shall turn out to be, of the functions of the brain, was no premeditated invention, but, like that of the principle of gravitation by Sir Isaac Newton, the result of accident. When he first observed at school that the boys who gained places from him by the facility with which they learnt and remembered words and recitations, while they were much inferior to himself in general talent, were all remarkable for a peculiar prominence of the eye, like that known by the name of bull's eye, he merely *remarked a fact;* and when he was removed to another school, and subsequently to college, his attention was arrested by the fact that there also the talent of learning easily by heart was accompanied by the prominent bull's eye. At that time he knew nothing of the cause of the prominence, nothing of the position, structure, or functions of the brain, and nothing of the philosophy of mind. He attempted no explanation, and had consequently no theory to support. He satisfied himself with observing that the fact was so.

For a long time Gall remained at this point; but, as he advanced in years and reflection, it at last occurred to him *that if one marked quality of mind was thus indicated by a peculiarity of conformation, the same might be the case with others.* This was the prelude to all his subsequent examinations. He began to remark with care the different forms of head and differences of disposition and talent by which his companions were respectively distinguished. To facilitate his researches and ensure greater accuracy of observation, he now took casts in plaster of every remarkable head or forehead which presented itself; and by comparing the peculiarities of each with what he knew of the mental qualities of their originals, he gradually became possessed of a very interesting series of observations throwing additional light upon the facts with which he started. Occasionally, when he thought he had succeeded in tracing a connexion between some marked feature of mind and peculiar form of head, an instance would present itself of the same mental peculiarity with a different form of head, and dash to the ground the conclusion which seemed approaching to certainty. Not discouraged by these results, he neither hesitated to give up the opinion which was thus disproved by facts, nor found his faith in the uniformity of nature at all shaken. He submitted to the correction, but continued his observations, and rarely failed by perseverance to discover the cause of his error and to add to the stock of positive truths. The ultimate result of his labours was the gradual development of the physiological and psychological doctrines now known under the name of phrenology.

Phrenology then may be considered in two distinct lights: first, as an exposition of the functions of the component parts of the brain; and secondly, as a theory of the philosophy of mind. Considered in the former light, the evidences of its truth must be sought for in oft-repeated observation of the concomitance and connexion of certain functions with certain portions of the brain; whereas, considered purely as a system of mental philosophy, its truth may be judged of, like that of other theories of mind, by the facility and consistency with which it explains the phenomena and admits of practical applications to the purposes of life. The former kind of evidence, viz. that of *direct observation*, is by far the most conclusive, and, as coming within the strict province of physiology, is that to which medical men ought chiefly or first to direct their attention. But the evidence arising from complete adaptation to the phenomena is also entitled to great weight, and may indeed suffice for those who study it chiefly as a branch of philosophy. The best way of all, however, is to investigate the subject from both points of view, and embrace both kinds of evidence; but on the present occasion we must confine ourselves almost exclusively to its consideration as a branch of physiology.

Taken in its widest sense, phrenology professes to be *a theory of the philosophy of mind, founded on the observation and discovery of the functions of the brain, in so far as that organ is concerned in the mental operations.* Its fundamental principles are the following:

First. That the brain is the organ of the mind, and is concerned in every mental operation, whether of emotion or of intellect.

Second. That the brain does not act as a unit, but consists of a plurality of organs, each serving for the manifestation of an individual faculty of the mind.

Third. That the energy of function or power of manifestation is proportioned, *cæteris paribus*, to the size of the organ; or, in other words, that a large organ will, *all other conditions being equal*, enjoy a power of action proportioned to its size, and consequently manifest the corresponding faculty with greater energy than if it were small.

And lastly. That by observing carefully a sufficient number of cases in which the same part of the brain predominates in size over all the other parts, and ascertaining what particular quality of mind is exclusively in excess in the same individuals, we obtain a direct clue to the discovery of the functions of all the organs of the brain, and require only that the observations shall be so carefully made and so extensively repeated as to obviate every chance of error before adopting the inferences as established. Let us now see how far these principles are in accordance with nature and with previously existing knowledge.

That the brain is the material organ, without the intervention of which the mind cannot operate during life, is so all but universally admitted, that we shall adduce no facts to prove it. It is true that some over-scrupulous men, like Lord Jeffrey and Dr. Abercrombie, still doubt whether the mind acts through the medium of material organs, except in its communications with the external world; but as the proposition is regarded by an overwhelming majority of physiologists as demonstrated, we shall, on the present occasion, assume it to be true.

Nearly the same assumption might be made with safety as to the brain consisting of a plurality of parts, each performing a distinct function. But the truth of this principle is put beyond a doubt by a mass of evidence which we cannot stop to detail, and is further confirmed by the successive additions which the brain receives as animals rise in the scale of intelligence, and by the successive development of its different parts, as the human being advances from the fœtal to the mature state, and from a state of unconsciousness to one of sensation, emotion, thought, and action. During this transition, the different parts of the brain are developed, not simultaneously, as a unit would be, but successively and irregularly. In one individual, eminent for talent, the anterior lobe is early and largely developed, while in another, whose intellect is purely idiotic, it remains small and contracted. In like manner, partial insanity, and injuries of the brain attended with a partial affection of the mental powers, equally afford a presumption of a plurality of cerebral organs. If necessary, it would be easy to multiply such indications and proofs; but as the advocates of the unity of the brain are few and far between, and their views are entirely without influence on the thinking part of mankind, we consider it needless to occupy more time and space in proving what is so rarely and feebly denied.

The *third* principle, and that which it is of most consequence to explain and demonstrate, is the proposition that *organic size is, cæteris paribus, a measure of functional power.* The first two principles are common to phrenology and to physiology in general; but the third, in its broad and specific form, is peculiar to and lies at the very foundation of phrenology, and will therefore require a more detailed and careful examination. If it be false, phrenology must crumble to dust like the dry leaves of autumn driven along by the winter's blast. If it be true, those who oppose phrenology on the assumption of its falsity must themselves fall, and like decaying leaves around the living parent stem, even serve to nourish and support that which they attempt to destroy. To the examination of this point we shall therefore, without scruple, devote considerable space.

The form in which the above principle is generally expressed by phrenologists is, that *size of brain is, cæteris paribus, a measure of mental power.* Inattention to the simple meaning of this proposition has been the chief cause of the opposition it has encountered from scientific as well as unreflecting men. Notwithstanding all that has been done by phrenologists to enforce attention to the important condition of "other circumstances being equal," almost all the opponents, from the Edinburgh Reviewer down to Dr. Holland—the latest who has published on the subject—continue to utterly disregard it, and speak of the proposition as maintaining that *size alone* is the measure of functional power; or, as Dr. Holland chooses to state it, that "*the gross condition of quantity represents the intensity of quality.*" Having set up this phantom of their own imaginations, like a pyramid on its apex, many of the anti-phrenologists proceed with heavy blows and an approving conscience to knock the support from under it; and when it topples over in obedience to their efforts, they turn round in triumph, and claim the merit of having upset *phrenology.* We have seen this feat performed again and again in the presence of phrenologists. On such occasions their simple answer was, "You have upset a phantom of your own creation, but you have left the phrenological pyramid, resting on its basis, untouched and undamaged;" and such is in reality the case.

As it is in general far more easy to make merry with fiction than with truth, it required no great effort of wit in Lord Jeffrey to divert his readers, by referring to grandmamma Wolf, in the fairy tale, as a high physiological authority on the side of the phrenologists, when she tells little Red Riding Hood that she has large ears to hear her the better, large eyes to see her the clearer, and a large mouth to gobble her up with the greater facility. But his mirth did not alter the substantial fact established by the researches of comparative anatomists, that where great nervous sensibility is required, whether for hearing or sight, a proportionally large nerve is an invariable accompaniment, whatever the shape or appearance of the organ on which it is ramified. Neither did it alter the fact that the venerable lady's large external ear was really capable of receiving a larger number of atmospherical pulses, and her large eye a greater number of the rays of light, than a smaller ear or eye would have been. His joke nevertheless was a good joke. It possessed the rare merit of diverting, at the same moment, not only himself and those whom he misled, but also those against whom it was directed. The only difference was, that he laughed at what he supposed the absurdity of his opponents, while they were merry at the absurdity of the egregious blunder into which he had fallen, and from perceiving that, in point of both fact and argument, the venerable grandmamma had the great reviewer entirely at her mercy.

If the phrenologists are to be judged by their own statements and acts, and not by those falsely ascribed to them, we should say that, so far from having adopted the proposition which Dr. Holland refutes, they even deserve credit for adding to the evidence formerly existing, that "gross quantity" or size alone is NOT a measure of the functional power of an organ. We have taken some trouble to enquire, and have never met with one phrenologist who did not utterly scout the notion of organic size being THE ONLY condition of functional energy; and who was not prepared with *proofs* by the dozen of the absurdity of such a proposition. Dr. Holland says, "this *relation of mere bulk of substance to the perfection or intensity of a faculty is, primâ facie, very improbable.*" To be sure it is; but what surprises us is, that a man of Dr. Holland's good sense should have had any doubts about the matter, when he might have satisfied himself of the fact by half an hour's observation; or, if he preferred the authority of others, by consulting any good phrenological treatise in his library. Yet, strangely enough, while he stickles about the insufficiency of the evidence in support of phrenology, he does not hesitate to admit opinions unfavorable to it upon no evidence at all; and in this particular instance really argues against one of its plainest and most easily demonstrable principles, merely because he has not taken the trouble to understand its meaning.

For demonstrative evidence of *organic size being*, cæteris paribus, *a measure of functional power* (a very different proposition from "mere bulk" bearing a constant relation to "intensity of quality"), we would refer the reader, first, to personal observation in the field of nature; and secondly, to the concurring testimony of every anatomist and physiologist who treats of the relation between structure and function. We are not aware of a single work of any reputation in which the above principle is not tacitly adopted as nearly self-evident. It pervades every corner of comparative anatomy, and is constantly, though not ostensibly, resorted to as a guide to the discovery of function. If, in an unknown animal, the optic nerve is found to be large relatively to the other nerves of the senses, we never hesitate to infer that the power of vision will be greater in proportion than where the nerve is relatively small. In the same way, we never discover a large olfactory nerve and extended nasal apparatus, without inferring that the animal must be endowed with a powerful sense of smell. And when it is affirmed by phrenologists, that the brain forms no exception in this respect to the rest of the organization, they merely state a principle in words which is admitted universally in practice. Indeed, all the modes of discovery hitherto employed, Camper's facial angle among the rest, tacitly assume this very principle as their basis; while it has been left to Gall and his followers to direct attention to it, and demonstrate its importance, as a specific truth. In proof of this statement, it would be easy to multiply quotations from any accredited work on comparative anatomy; but one from an indisputable authority may suffice: "It appears," says Cuvier, "that *there are always certain relations between the faculties of animals and the* PROPORTIONS *of the different parts of the brain.* Thus, their intelligence appears to be always great in proportion to the development of the hemispheres and their several commissures. It appears even that *certain parts of the brain attain*, in all classes of animals, A DEVELOPMENT PROPORTIONED *to the peculiar properties* of these animals; and one may hope that, in following up these researches, we may at length acquire some notions respecting the particular uses of each part of the brain." On another occasion, when speaking of the cerebral lobes being the place "where all the sensations take a distinct form, and leave durable impressions," Cuvier adds, "l'anatomie comparée en offre une autre confirmation dans *la proportion constante du volume de ces lobes avec le degré d'intelligence des animaux*;" thus admitting the influence of *size* of the cerebral organs upon the power of manifesting the mental faculties as distinctly as Dr. Gall himself could assert it.

But, it may be asked, if the principle of size being, *cæteris paribus*, a measure of power, has been thus virtually and universally admitted by men of science, whence arise the objections advanced against it by such men as Dr. Holland, when it is *specially* brought forward by the phrenologists? The only answer that can be given is, that the full value of the principle as a means of successfully prosecuting enquiry, was unknown till demonstrated by Dr. Gall, and that consequently it had never been a subject of serious consideration among men of science as a distinct and specific proposition. Even now, however, its truth is so palpable that it is never objected to, except when confounded with the very different and erroneous proposition that size *alone* is a measure of power; and, in point of fact, Dr. Gall has been the first to explain the apparent anomalies which other physiologists met with in their researches, by drawing attention to the necessary limitation of *cæteris paribus*. And when this is kept fairly in view, it becomes nearly as impossible to deny it, as to deny that a whole is greater than a part. Both phrenologists and antiphrenologists are agreed, for example, that a large forehead *generally* indicates superior intelligence; but the faith of the former in the influence of organic size, as affecting intensity of function, is not in the least shaken by the fact that there are *some* large foreheads unaccompanied by any intellectual superiority. Nobody, indeed, knows this fact so well as the phrenologist, because he has not only observed it, but alone has examined the cause of the difference, and found that *the other conditions of the brain are not the same*, and, consequently, that so long as cause and effect continue related as such, the results in mental power cannot possibly coincide. The large and healthy expanse of brow which distinguishes the bust of Bacon may be equalled, *in mere size*, by the unhealthy expanse of forehead in the cretin or idiot; but will any one venture to infer from this that the size of Bacon's *healthy* brain added nothing to its functional power? A single example of this kind is sufficient to demonstrate that size alone is not a measure of intensity, but it leaves absolutely untouched the phrenological proposition that size is an important condition of functional power. Great energy of mind cannot coexist with a small size of brain, because no other healthy conditions can supply the want of size. But a large brain may coexist with feebleness of mind, because from original malformation, defective constitution, or disease, its power of action may be also defective. Large muscles, in the same way, may coexist with little bodily strength in a very lymphatic or relaxed constitution, and in certain states of health; and yet it is never doubted that, *all other conditions being equal*, large muscles are more powerful than small ones. For more than this the phrenologists do not contend.

Had Dr. Holland attended to the foregoing most obvious distinction, as laid down in all the works on phrenology which we have ever seen, he would scarcely have ventured to misrepresent Gall's discovery as resting "on the presumption of the gross condition of quantity representing the intensity of quality;" and, when speaking of the small brains of idiots, and the large brains of eminent men, as affording the best proofs of the influence of size, he would have had no difficulty in explaining the *apparent* exceptions to which he alludes, and reconciling them to the general rule. Rightly interpreted, *there can be no exceptions to a law of nature;* and when we meet with cases which seem to contradict the principle of organic size being a chief condition of functional power, we can come only to one of two conclusions. Either the principle *must be fallacious* and size be wholly uninfluential *in all cases*, or it *must be real and operating in all.* In particular cases its power may be controlled or its action modified, by causes which have escaped observation; but there is no contradiction in the laws of nature, and we may rest assured that if the principle under discussion has a real operation in any case, it will exercise an influence in all, whether or not we can detect the causes by which its perceptible results are modified.

We almost feel that an apology is due to our readers for insisting so much on so obvious a truth; but the very fact that science has been retarded by its neglect and misconstruction, compels us to enforce it even at the risk of tediousness. Sometimes in conversation, after we imagined that the question was placed clearly before the mind's eye, we have been met with the triumphant assertion that our proposition was annihilated by the simple comparison of the small brain of the intelligent poodle with the large brain of the stupid ox. But are all the other conditions the same in such a case except size? No doubt the brain of an ox is a brain as well as that of a poodle; but is there no difference in their structure, no difference in the proportions of their anterior lobes, and no difference in the number and complexity of their convolutions sufficient to exercise an influence on their functions in addition to mere size? Looking to the philosophical principle of *cæteris paribus*, it is clear that the proper way to arrive at the truth is to compare the brain of a clever with that of a stupid poodle, and of one ox with another, as nearly as possible of the same age, state of health, and constitution. If this be done, and the intelligent poodle be found to have the smaller anterior lobe, then by all means denounce the principle of size as untrue and at variance with fact. But if the reverse be the case, do not attempt to set the truth aside, by comparing two things so essentially different as to make absolute agreement impossible. If this precaution be kept in view, we venture to affirm that the more the proposition is scrutinized, the more firmly will it be found to rest on the unassailable foundation of truth.

Admitting the brain to be the organ of the mind; admitting also that the brain is not a unit but a congeries of organs, each having its appropriate and peculiar function; and lastly, admitting that the energy of every function is proportioned, *cæteris paribus*, to the size of its individual organ; it follows necessarily, as is remarked by Cuvier, that the size of any cerebral organ affords a direct clue to the discovery of its function. Let us suppose, for example, that the use of the optic nerve was unknown, but that it was invariably found to be far more largely developed than any of the other nerves of sense, in animals with powerful vision; such as the eagle, and much less so in animals which see very imperfectly, such as the mole; and that no instances were to be found in the same species, in which, *all other circumstances being equal*, powerful vision coexisted with the smaller nerve, or a larger nerve with feebler vision; would we not be justified in at length inferring that the use of the nerve was to serve for vision? In like manner, if a particular portion of the brain is invariably found to be large, in relation to the other parts of the same brain, in individuals remarkable for timidity and wariness, and relatively small in persons remarkable for rashness and the absence of fear, and no instance can be adduced in which, *cæteris paribus*, the proportion between the feeling and the organ is reversed, are we not entitled, after sufficiently extensive observation, to hold that the use of that part of the brain is to serve for the manifestation of the sentiment of cautiousness? And if this mode of investigation is applicable to one part of the brain and to one faculty of the mind, it is obviously applicable to all. The only indispensable condition of evidence of this description is that the coincidence shall be real and uniform, and not imaginary or accidental; and here is precisely the grand point of difference between the phrenologists and their opponents, and in regard to which the former have never been fairly met. But as this point is of fundamental importance in determining the truth of phrenology, it will be necessary to devote a little space to its consideration.

The phrenologists affirm that by observing concomitance of function with size of organ in an infinite variety of instances, as above explained, they have succeeded in tracing a connexion between certain faculties of the mind and certain portions of the brain. Whether there are sufficient grounds for maintaining the existence of such a connexion is evidently a *question of fact*, against which *à priori* argument can be of no avail. The only way to meet the phrenologists successfully is to adduce facts at variance with their conclusions; and even Dr. Holland admits that the conclusiveness of this appeal cannot be denied, for he allows that if the facts tally with the statements of the phrenologists in a large proportion of cases, so as to make reasonable allowance for error or ambiguity, *the improbability must be laid aside, and the whole admitted as a new and wonderful truth.* "Here, then, by common admission, is a direct question of evidence, the amount and strictness of which are solely to be considered."

Dr. Prichard, and other writers on the same side, take a similar view of the subject; but the phrenologists complain, and not without reason, that the very men who are foremost in admitting the question to be one of fact alone, are the first to "turn their backs upon themselves," and attempt to solve it by argument and probabilities, which, considered *as evidences*, are worth nothing. Instead of meeting the followers of Gall by well-observed and hostile facts, Dr. Holland merely says, "Here I think it will be found that the phrenologists are yet wanting in what is needful to establish their system, notwithstanding all the observation and ingenuity which have been bestowed on its proof;" and in answer to their facts, he contents himself with assigning sundry reasons for quietly setting them aside.

"Look," he says, "at what they have in aid of their determinations, where the question concerns the relation between a certain outward form of cranium and some faculty or quality of mind, alleged to be in correspondence with it. First, the equal chance of affirmative or negative, as to each particular quality predicated. Secondly, the plea of a balance of some indications by others and opposing ones. Thirdly, the want of exact definition of many of these qualities or faculties making it difficult to arrest for error where there are so many ways of retreat. And fourthly, the incidental discovery of character by other and more ordinary methods. I well know that the candid disciples of the system will not consciously avail themselves of all these methods. Nevertheless, each one of them has, more or less, been made use of; and looking to the chances and facilities thus obtained, it may be affirmed that the number of true predictions in phrenology is less miraculous than it would be, were this number not to exist." (p. 509.)

We admit at once that all this is very plausible, and that, as a reason for exercising caution in observing and in drawing inferences, it is very useful; but *does it in any degree meet the question of fact, and prove that the alleged coincidences are unreal?* We cannot see that it does, and we are of opinion that one well-authenticated fact, opposed to those of the phrenologists, would outweigh a volume of reasoning in a matter of this kind. Dr. Holland states that phrenologists appeal to coincidences between mental power and cerebral development, but he regards the coincidences as "not sufficiently numerous," and adds that during his intercourse with Gall and Spurzheim, he had several opportunities of noticing the failure of their judgments upon these particular faculties, as well as in other cases where the doctrine ought to have indicated rightly the relation between faculty and organ. But Dr. Holland does not adduce any details of these failures from which his readers might judge for themselves, whether they were real, and if so, whether they resulted from the outward indications being erroneous, or from a mere personal blunder in estimating them, such as may happen and does happen daily in the case of a chemist or mathematician, whose science nevertheless remains unaffected by the blunder.

We also have heard of erroneous inferences being made by phrenologists, and have taken some trouble to investigate their nature. In some, we should say in most, instances, the error has proceeded from the rash judgment of incompetent persons. In others, we have known a well-qualified phrenologist commit a mistake, either from giving an opinion hurriedly, or from speaking more decidedly than the real difficulties of the case warranted. There are instances, for example, in which a number of organs are so equally developed, and in which the corresponding mental powers are so nearly equal in energy, that it is impossible to assign a marked predominance to any of them. It is in cases of this kind that the influence of education and external circumstances is greatest, and that the quality which is most assiduously cultivated will assume prominence in the character. Take two men, for example, in whom the selfish and the devotional feelings are originally almost equally strong, and breed the one to the church, and confine him to the society of the kind and benevolent, while you place the other in a counting-room, amidst all the excitement of money-getting—the one will assuredly become, not pious and disinterested in the highest degree, but certainly *more* pious and disinterested than the other; while the phrenologist, who affirmed that they were naturally or originally on an equality in this respect, and that the two faculties were nearly equally balanced in both, would most likely be regarded by their respective acquaintances as greatly in error. Again, we have known a phrenologist hastily pronounce an organ to be moderate, which was really large, and thus give rise to an apparent contradiction. But although this may happen now and then, *it does not alter the reality;* it leaves the organ of the same size as before, and if a more careful comparison shows it to be really large, the induction remains valid, although the manipulator committed a mistake. This, however, is carefully kept in the back ground by the opponents of phrenology, who often confound an erroneous estimate of a fact with hostility of the fact itself, and thence infer that phrenology must be in fault, when there has been merely an error on the part of the individual, for which the science ought never to be made answerable. If the observations made by the phrenologists *are* incorrect, surely there can be no great difficulty in obtaining authentic facts to prove their inaccuracy. And yet, while all thinking men on both sides agree that the question can be authoritatively settled only by a reference to fact, it is somewhat remarkable that the phrenologists alone have taken pains to observe nature and to form collections of facts, which they have further laid open to public inspection and verification in their museums; while their antagonists have neither published nor collected any opposing facts, but have contented themselves with the vague assertion that such exist, and with arguing that therefore those of the phrenologists must be untrue.

Here, we think, lies the great error of those who contend against the truth of Gall's discovery. All of them—even Dr. Roget, Dr. Prichard, and Dr. Holland—state, *in a general way*, that their experience is against the alleged concomitance of mental faculty and cerebral organ. But instead of themselves specifying facts and giving details entitled to confidence, they complain that the observations recorded by the phrenologists are not "sufficiently numerous" or accurately made to prove their positions, and argue that hence these must be disbelieved. This mode of proceeding, when expressed in plain language, appears palpably absurd. The phrenologists state principles, and adduce "some" facts patent to everybody, which tend, *pro tanto,* to prove them. Their opponents, however, say, "No; do not believe one of them, for *we* know facts which do not tally with them, but which *we shall keep to ourselves*, and which you must believe merely on our assurance." The phrenologists have been accused of claiming a large measure of belief on the part of their followers; but their claim is backed, not only by hundreds of published cases, but by museums full of specimens, copies of the more remarkable of which are to be found in almost every large town in Britain. Whereas the anti-phrenologists make a sweeping claim on the public to disregard all these evidences, and to believe them worthless on their own mere affirmation, unsupported by facts of any description! Is it to be wondered at that opposition so directed has been wholly ineffectual in arresting the progress of phrenology or disproving its truth? We think not; and we suspect that if phrenology is to be put down at all, it must be by an opposition more in harmony with the Baconian rules of philosophizing than any hitherto attempted.

Dr. Holland, Dr. Prichard, and Dr. Roget, all have the sagacity to perceive that, however plausibly the matter may be argued on either side, the truth of phrenology must in the end be decided by an appeal to facts alone; and such being the case, we think our remaining space will be much more profitably occupied with a few remarks on the best mode of testing the phrenological facts by observation, than with comments upon any other parts of the general argument.

If it were necessary, this would be the place to show that there are no insuperable difficulties in the way, to prevent the size and configuration of the brain from being pretty accurately estimated during life, by observing the outward form of the head. In the early days of phrenology, the want of parallelism between the tables of the skull, and the existence of the frontal sinus, used to be rather favorite objections. But they are now nearly abandoned by anatomists. Some parts of the skull are always thicker than others, but the greatest difference in the thickness of the parts, which have reference to phrenology, scarcely ever exceeds one or two lines, whereas, in cases of extreme development of brain, the difference of external size often exceeds an inch; so that, even after allowing for *the utmost possible* divergence between the tables, enough will still remain to indicate the development of brain below.

The existence of the frontal sinus generally makes it difficult in mature age, and especially in males, to ascertain the size of two or three of the smaller organs situated, according to the phrenologists, behind it; but we cannot see that it is of the least weight as an objection to the truth of phrenology in the main. The sinus rarely appears at all before puberty, and consequently cannot interfere with the accuracy of observations made before that age. It is also rarely much developed in females, and therefore an ample field for observation is open to which no objection of this kind can apply. But in this, as in other cases, the scope for controversy would be greatly narrowed, and truth be far more easily attained, if both parties were more careful to fix their attention principally upon the real objects of discussion, and not to lose sight of essentials in their keen pursuit of mere accessories, which serve only to perplex and mislead.

Admitting, in its fullest force, everything that can be said about want of parallelism between the tables of the skull, and about the existence of a frontal sinus of variable magnitude, all that we can honestly conclude is, not that the unsoundness of the phrenological principles has been established, but that a certain amount of difficulty stands in the way of their universal application. Thus, in some cases of chronic disease, the thickness of the skull increases to the extraordinary extent of an inch or upwards, and in other instances it diminishes to little more than the thickness of paper. In old age, also, the skull is sometimes of very irregular thickness, from the inner table following the surface of the diminishing brain faster than the outer. But during health and in mature age, such aberrations are never to be met with. When they do occur, however, it becomes evidently impossible to determine, *with certainty*, from the mere examination of the outward form of the head, the size and form of the contained brain; and therefore, Dr. Gall expressly *rejects, as inconclusive*, all observations made during *old age and disease*, because they necessarily involve an element of doubt. Many of such cases afford valuable *illustrations*, but can never be received as *proofs*. These must be derived exclusively from the period of life during which the essential correspondence between the external indication and the form of the brain can be relied upon.

In investigating the claims of phrenology, in short, it ought never to be forgotten, by either friend or foe, that the first and grand object ought to be *to ascertain its truth;* and that till this be done, it is needless to confuse the question by discussions referring solely to the difficulties of applying it to individual cases. The greater the facilities afforded for the verification of evidence, the sooner and more easily will phrenologists succeed in obviating all the difficulties of mere application; and if the balance of evidence shall turn out hostile, the matter will be at an end at once, and further discussion on any part of the question will become altogether superfluous and unnecessary.

How, then, are the alleged facts of the phrenologists to be most easily verified or disproved? As neither argument nor ridicule can set them aside, our only remaining, and by far the shortest, way is at once carefully to examine nature, and see whether our observations harmonize with or contradict those of the phrenologists. If they agree, let us give up prejudice and adopt them as true; and if they differ, let us at once reject them, and all the inferences deduced from them, as incorrect and untenable.

In surveying mankind, with a view to observe whether the alleged concomitance between certain qualities of mind and configurations of brain holds good, it will be apparent to every thinking enquirer, that a large proportion of society consists of what are called common-place characters, who are not distinguished by any striking mental feature of either a good or a bad kind, and who display an average amount of kindness, piety, conscientiousness, affection, pride, vanity, caution, selfishness, and temper, and also about an average amount of acuteness of perception and reasoning power; but who exhibit neither genius nor originality, and never seek to leave the beaten path of everyday usefulness in which Providence has placed them. On minuter examination, each individual of this large class is found to be distinguished by *shades* of the general character, and to possess a little more of one quality and a little less of another than his neighbour, but still to display nothing that marks him as very distinct from the general herd. If, as the phrenologists affirm, the development of the brain corresponds with the features of the character, it will follow that the mass of mankind, in any one locality, will present brains differing little from each other, and equally allied to a common type as we have seen their characters to be; but that, on minute examination, shades of difference will be perceptible in their heads, corresponding to the differences really existing in their minds. But it will also necessarily follow, that the difficulty of observing and appreciating these minuter shades of cerebral differences must, to an inexperienced person, be equally great, as it would be for a stranger to discover, at a first interview, the slighter shades of character by which each is distinguished from his neighbour.

Influenced by the difficulties of accurate observation amidst a general uniformity of this description, the phrenologists wisely advise beginners not to trouble themselves at first by looking for *proofs* among individuals known only for average mental endowments, and in whom, consequently, all parts of the brain may be nearly equally developed. After they have acquired experience in observation, they may obtain additional light by this means; but *in testing the truth* of the phrenological concomitance, it is far more satisfactory to begin with well marked cases, in which one or several of the mental faculties are very strong or very deficient, and in which, consequently, if phrenology be true, we may expect to find the corresponding parts of the brain equally remarkable for size or deficiency, and therefore easy of observation. For the same reason, they advise that the larger organs of the propensities or moral sentiments be selected for verification, in preference to the smaller and more difficult organs of intellect, and that the attention be fixed, at first, exclusively on strongly marked cases, in which no doubt can exist either as to the energy of the mental faculty or the magnitude of the organ. We would even go farther and counsel those not much accustomed to precise observation, to commence with cases in which a particular region of the brain or group of organs preponderates over the others, and in which the character is broadly marked by the energy of the corresponding faculties; just as in studying the geography of a new country, we should first make ourselves familiar with its leading features, and more general divisions into districts and counties, before seeking to determine minutely the positions of its towns or the precise courses of its rivers. When the eye is thus trained to the correct observation of the larger features, it will experience much less difficulty in taking accurate cognizance of details.

According to the phrenologists, the brain, considered as the organ of mind, may be divided into three great regions, the first comprising the anterior lobe, and serving for the operation of the intellectual faculties; the second comprising the coronal region, and more immediately connected with the moral sentiments; and the third comprising the posterior lobes and base, and serving for the manifestation of the propensities common to man with the lower animals. In a person of a well-constituted mind, these three regions, and the corresponding groups of faculties, are in due proportion to each other; but wherever the character is marked by the predominance of the lower passions and by feebleness of intellect and moral emotion, as in most criminals, the posterior and basilar regions will be found in excess, and the coronal and anterior portions narrow and defective, or the "forehead villanous low." Where, on the contrary, as in Melancthon, the moral sentiments and intellect form the prominent features of the mind, and the passions are weak, the anterior and coronal regions will rise high and arched over a comparatively small base and posterior region.

Here, then, is a good field for a beginner. To ascertain how far physiologists in general, as well as phrenologists, are right in considering the anterior lobe of the brain to be more immediately connected with the intellectual faculties, it will be easy to compare the expanse of forehead in congenital idiots with that of men of ordinary intelligence, and still more of men of great and general talent. In most cases of this kind, the idiocy arises from defective development of the brain, and especially of its anterior portion; and it requires one only to visit a few asylums or workhouses to observe the stinted dimensions of the foreheads of idiots, as contrasted with the lofty brow of a Bacon or a Shakspeare. The creative genius—the highest attribute of intellect—of Michael Angelo scarcely formed a more striking contrast to the mental inanity of the idiot mentioned in the Phrenological Journal, vol. ix., p. 126, than do their respective foreheads represented from nature in the annexed woodcuts.

Michael Angelo.

An Idiot, aged 20.

If we pursue this enquiry throughout the whole family of man, we invariably find the forehead most developed among the races most remarkable for general intelligence and the reverse. Lowest in the scale of organization are perhaps the aborigines of some parts of New Holland; and from them we have an almost regular gradation through the Carib, the Esquimaux, the North American Indian, the New Zealander, the Negro, the Sandwich Islander, and the Hindoo, up to the European, who has decidedly the largest forehead and highest intelligence of them all. It is true that among idiots we occasionally find an example of a very large and prominent forehead and head, as among the cretins of Switzerland; but these are generally cases of hydrocephalus, or of other forms of cerebral disease in which disorganization has taken place, and in which the mental faculties have become impaired as the disease advanced. We have seen smallpox induce idiocy in this manner in a scrofulous subject; and it is not an uncommon termination of long-continued mania. These facts, however, constitute no exception to the axiom, that a brain below a given size is incapable of manifesting the mental faculties in a healthy and efficient manner.

If, to induce us to test the fact by direct observation, we required farther presumptive proof of the connexion of the anterior parts of the brain with the intellectual powers, we would refer to the general experience of mankind, and to the many attempts made to measure the one by the other. Camper's celebrated facial angle, which affords results generally accurate but presents easily explicable exceptions, is founded on the principle of the anterior lobes being not only the seat of intelligence, but proportioned in development to the extent of the intelligence; and it fails only from overlooking disturbing causes, which phrenology at once points out, and enables us to avoid.

To ascertain the connexion of the animal propensities with the posterior and basilar portions of the brain, we have only to observe the heads of men who are notorious for the fierceness of their passions, for selfishness, cunning, and utter want of principle; and those of men whose delight is in doing good, quietly and unostentatiously, and whose passions are never roused, except in defence of suffering humanity. If, for example, the heads of a Sykes and a Fagin do not form a contrast, in the preponderance of the basilar regions, with those of men like the Brothers Cheeryble of Boz, as remarkable as the contrast between their characters, we need scarcely go further, as it would prove, unexceptionably, the non-existence of the alleged concomitance. But if the contrast is, in reality, as striking as it is said to be, then let us note it well, and continue our observations on characters of a different kind, till evidence shall accumulate sufficient to warrant an opinion on the general truth of the principles on which the phrenological mode of investigation is founded.

Having ourselves bestowed much pains on the verification of the phrenological evidence, and learnt, by experience, the best way of surmounting its attendant difficulties, we would earnestly recommend those of our readers, who are really desirous of satisfying themselves of the truth of Gall's discovery, to begin by visiting any of the phrenological museums, such as the splendid collection of Deville, in the Strand, and placing, side by side, thirty or forty heads of abandoned criminals, and as many of persons of superior intelligence and morality, and contrasting the general features of one class with those of the other. In this way, differences will become palpable, which, viewed singly, might be overlooked; and if, with shades of difference in other respects, the whole of the criminals' heads shall be found to present a large base of the brain, and a comparatively low and narrow forehead and coronal surface, while those of individuals noted for superior virtue and intelligence show the proportions reversed, it will become very difficult to deny the probability of some fixed relation subsisting between the organization and the mental qualities. We have tried this test, on a great variety of occasions, in France, in Italy, and in Germany, as well as in this country and in Ireland, and we feel bound to admit, that the general coincidence was very striking. Among the criminal heads we found two or three, on different occasions, which presented a larger forehead and coronal development than the rest, and which brought them nearer the type which is considered to indicate average morality and intelligence; but, on further enquiry, we found that these apparent exceptions belonged to criminals superior to their class, by the very traits of character which their heads indicated; and that they had come under the law of the land, not from the energy of low and brutal passions, but from employing their intellects in schemes of embezzlement or forgery. We are not aware, however, of even one instance of a really ferocious and degraded character being unaccompanied by a decided preponderance in the basilar and posterior convolutions of the brain. Nor have we been able to discover a single example of a person presenting such a development, being noted in the world for refined morality or elevation of mind.

Having repeated this experiment a sufficient number of times, with different sets of heads, it will be instructive next to compare the skulls of savages with those of any of the European nations, or the least civilized with the more civilized—the New Hollander, for instance, with the Hindoo, or the Carib with the South Sea Islander. The Phrenological Society of Edinburgh possesses, what Tiedemann, after visiting it, admits to be the largest existing collection of national skulls, and many of the societies scattered throughout the kingdom possess either skulls or casts of skulls from various parts of the world. Deville's museum also contains many, which are accessible to every one. In several of these museums we have tried the same plan of contrasting different races with each other, and, speaking generally, the coincidence of development of brain, with the known character of the respective races, appeared such as could hardly fail to strike every intelligent and conscientious observer, as affording the strongest presumptive proof of Gall's discovery. We introduce the annexed diagrams as strikingly illustrating the foregoing observations and our subject generally.

Negro

Hindoo

Peruvian.

European.

Sandwich Islander.

The Poet Burns.

We have seen an exhibition of national skulls arouse attention and excite an interest, which ended in ultimate conviction, in minds prejudiced to the last degree against phrenology; and it may be thought worthy of notice, that the anatomist Dumoutier, who is the Deville of Paris, is at this moment on a voyage round the world, in one of the discovery ships sent out by the French government about a year ago; and that the principal object of his mission is to collect skulls, and take casts or drawings of the skulls and heads of the natives, wherever the ships may touch, for the purpose of serving as phrenological illustrations. We have no doubt that he will return with a rich and valuable collection. In this respect, the conduct of the French government differs widely from that adopted by our own about ten years ago, when the collection of skulls, made for phrenological purposes, by Mr. Collie, Surgeon of H.M.S. Blossom, during a similar voyage of discovery, was taken possession of on his return, and rendered of no use either to science or to himself. Captain Beechey would not even accept the offer of a short report on their phrenological indications, which was volunteered by Mr. George Combe, and which would have added to the interest, at least, of Captain Beechey's narrative, without possibly doing it any injury.

Having so far prepared himself for making accurate observations, the next step for the phrenological enquirer will be, to examine the general outlines of the heads of those persons whose dispositions are most marked and best known to him; still confining himself, however, to the regions rather than to individual organs. Let him for a time disregard all medium cases, and seek only for extremes. It is from the latter that PROOFS are to be most satisfactorily obtained; for, as yet, the numerous difficulties inseparable from imperfectly defined cases, would only perplex and confound him. The medical man possesses many advantages in pursuing this enquiry. He not only sees human character and human weaknesses in the confidential intercourse of private life; but in hospitals, in gaols, and in schools, he may select the most conclusive cases as evidence, and multiply proofs to his heart's content, before pinning his faith to any man's creed. But in all his proceedings, let him be cautious and steady; neither hasty in adopting evidence, nor precipitate in rejecting it. Some things appear at first sight to be conclusive, for or against a doctrine, while they are so only from being imperfectly known. But wherever, on due examination, facts seem to demonstrate a truth, let nothing turn him away from its adoption; and, on the other hand, let nothing tempt him to retain an opinion which facts appear conclusively to falsify.

It will be found impossible, we think, for any candid person to pursue the above mode of enquiry, for any considerable period, without becoming impressed with the conviction, avowed by other eminent observers as well as by Gall, that the degree of intelligence is, *cæteris paribus*, proportioned to the development of the anterior lobes of the brain, not in man only, but also in the lower animals. In Vimont's magnificent work on Comparative Phrenology, proofs of this fact superabound; and it is a matter of common observation, that dogs, horses, monkeys, and other animals remarkable for intelligence, have large and rounded foreheads. We are aware, indeed, of supposed exceptions to the rule in persons who present an apparently large and broad forehead, and yet are by no means superior in talent. But in all such cases, where the original constitution or temperament is not very low, and disease has not impaired the cerebral functions, the anterior lobe will be found to be really very moderately developed; and the fallacy to arise from judging of its size by height and breadth alone, *without taking depth into account*. A deep anterior lobe is one which extends far forward over the orbitar plate of the frontal bone, and projects over the eye and cheek-bones. A shallow anterior lobe, on the contrary, is short, and scarcely advances far enough to protect the eye. The distance forward, from the lower extremity of the coronal suture, is a good indication of the length of the anterior lobe, and will be found to vary not a little, even where the mere fronts look equally large. This will be easily understood, by supposing an observer to be placed directly opposite the ends of two logs of wood, each a foot square, but the one twenty feet long, and the other only ten. It is clear, that were he to judge merely from the end view, he would declare both logs to be equal, although, in reality, the one was double the size of the other. It is the same with the anterior lobe; in order to avoid mistakes, its depth or length must be reckoned, as well as its height and breadth. We have heard this called a "loop-hole" for the phrenologists; but, call it by what name you please, the question which concerns us is, simply, whether it is *a fact?* We confess that our experience obliges us to admit the reality of the distinction here pointed out, although at one time we overlooked it; and it is nowhere more palpably seen than in the large-looking but really shallow foreheads of the Peruvian skulls, compared with the apparently smaller but much deeper foreheads of the Greeks, French, or British.

A similar precaution is required in estimating the development of the coronal region of the brain. Many of the criminal heads present a rather broad upper surface; but it extends almost like a flat plain, and rises little above the level of the points of ossification in the parietal and frontal bones, instead of forming the high and arched appearance which we remark in the heads of Sully, Melancthon, and others, noted for the energy of their moral feelings. But the best way to ascertain the real size of the coronal region, is to compare a number of heads of persons remarkable for moral endowments, with those of depraved criminals, or of persons known to be deficient in the higher feelings of our nature. If this plan be followed, the difficulties will, to a great extent, disappear. But in this, as in other comparisons of a similar kind, it ought to be kept in mind, that it is not the absolute size of a portion of the brain, in one individual, that is to be compared with its absolute size in a different individual. The true point of comparison is the predominance of a given portion over the other portions *in the same head*, with a similar preponderance over the other portions in a different head. The comparison is, therefore, not a single but a double one; and it is not absolute size that is to be compared, but the relation between an existing preponderance in each of two heads, considered with reference *each to its own standard.* For example, there is a wide difference betwixt affirming that A's nose is larger than B's, and affirming that A's nose is larger relatively to the rest of his features than B's relatively to his. The latter proposition may be perfectly correct, and yet B's nose be the larger of the two in absolute size. It ought, therefore, to be distinctly understood, that in all comparisons between different heads, this double standard or comparison is implied—because, if this be overlooked, much confusion may arise.

Having thus made ourselves familiar with the larger divisions of the brain, we next proceed, in our verification of the phrenological evidence, to test the functions ascribed to the individual organs or portions of the brain; and here, also, every precaution must be used to avoid error, and we should be careful to begin with those organs, which, from their size or situation, are most easily observed.

Some of the leading propensities are in great activity in childhood and youth, and, when possessed in a high degree, present very favorable opportunities to the enquirer. In early life, manners are not yet broken into that conventional standard to which most people endeavour to approximate on becoming active members of society, and consequently the natural qualities of the individual stand forth in a more recognisable form than at a maturer age. Hence the facility with which we may then test such propensities as self-esteem, the love of praise, cautiousness, affection, secretiveness, and destructiveness. The sly timidity and shyness of one child contrasts strongly with the bold and confident openness of another. In one, a fiery temper rages without control; while another is remarkable for patient submissiveness. Contrasts such as these cannot be mistaken, and if the organization shall not be found in harmony with each, phrenology must inevitably perish. Facts alone are what it has to stand upon.

It would be out of place, even were it possible, to enter here into a detailed exposition of the mode of observing every individual organ, or of the evidence on which its function is held to be ascertained. For that we must refer to the works named at the head of our article, and particularly to the "Functions of the Brain" of Gall, the "System" of Combe, the "Human and Comparative Phrenology" and plates of Vimont. All that we can do here is to point out such things as we found most useful in making our own observations, and to add that in verifying the individual organs, we derived the greatest assistance from placing side by side (but always with reference to the principle already explained) heads and skulls in which the organ in question was possessed in opposite degrees of development. Thus, in examining destructiveness, we placed a row of murderers and ferocious savages alongside of a row of virtuous characters and Hindoos; and in studying the organ of tune or melody, we contrasted a row of musicians with an equal number of persons indifferent to music. In this way, the larger features come out prominently, and leave no doubt as to the conclusions deducible from them. It is in this way that the collections of skulls and casts of dead and living characters, formed by Deville and many of the phrenological societies, become of great practical value; and we would advise those who, like Dr. Holland, reject the evidence altogether, on the plea that the facts are not numerous enough, to study for three months those which *already* exist in such a collection as Deville's, before they again express an opinion on the subject. We are far from thinking that, after doing so, they will agree in every inference drawn from them by Deville himself, or by other phrenologists; for the latter, like other fallible men, often enough take a step beyond the point of solid support, and in consequence sink into the mud of error. But we should be greatly surprised to meet with any man of average honesty, intelligence, and industry, who did not rise from such an enquiry with a higher respect for the genius and labours of Gall, and with more than a suspicion that the new physiology of the brain is true, in its great principles at least, and requires only to be assiduously cultivated to lead ultimately to a rich harvest of important results. To those who really seek truth, we would say, Do not be too much influenced, either by the successes or the failures of the phrenologists, but *go to nature and observe for yourselves*. Individuals may make "lucky hits" or occasional "mistakes;" but if the main facts are true, they will remain to speak for themselves, in a voice which cannot be misunderstood by any one desirous of understanding them; and will be found to substantiate the opinion of Cuvier—that, as "*certain parts of the brain attain, in all classes of animals, a development proportioned to the peculiar properties of these animals*, one may hope, *by following up these researches, at length to acquire some notion of the particular uses of each part of the brain.*"

Before leaving this part of the subject, we must repeat, that in judging of the development of an individual organ, as a direct test of its function, *its size ought first to be compared with that of the other organs in the same*

*head*, and not with any abstract or ideal standard. A faculty is strong or weak in proportion to the other faculties of *the same* mind, and the general character takes its hue from *its own* predominant qualities. Hence the obvious necessity of measuring mental power and cerebral development with reference to the individual himself, when seeking for *proofs* of the concomitance of the one with the other. It is only by keeping in mind this standard, that we can compare the size of an organ in one head with its size in another.

Long as we have already dwelt on the subject, there are numerous points of much importance, directly connected with it, which we have been obliged to pass over in silence, and others which we have touched upon very cursorily. But as our object is not to teach phrenology, but to draw attention to it as eminently deserving of serious enquiry on the part of the profession, our omissions are of less consequence. At the same time, we wish we could have spared room to state more fully what phrenology is, and to show a few of the numerous applications which may be made of it if it shall prove to be true. In the prevention, discrimination, and treatment of insanity and of nervous diseases, it already affords great assistance to the physician; and when it shall be freed from some of its accompanying errors, and brought to a maturer state, there will hardly be a possibility of overrating its practical value in education, in legislation, in the prevention of crime, and the treatment of criminals, as well as in medicine. If true, it furnishes the elements of the physiology of the brain and of the philosophy of mind; and no ghost is required to tell us how useful both of these branches of knowledge must be in improving mankind, and adding to human happiness. Although we are not so thoroughly satisfied as to consider ourselves phrenologists, in the full sense of the term, we have paid enough of attention to it, to warrant our forming a high estimate of its value, if it shall ultimately prove to be true. That it is rapidly advancing in professional estimation, is evident from many signs, and, perhaps, from none more clearly than the extent to which our best-conducted lunatic asylums are already under phrenological guidance. Every day, indeed, is adding to the number; and the direct evidence, proceeding from many quarters, that phrenology is found of daily and hourly use in the treatment of the insane, certainly affords a strong presumption that, in its great outlines at least, it must be both true and valuable.

We have said nothing about the objections against phrenology, founded on its alleged tendency to materialism, fatalism, irreligion, &c. &c.; because discussions about consequences are utterly superfluous till the truth be ascertained. IF PHRENOLOGY IS A TRUTH, *it is impossible that its use can lead to anything bad.* If it is true, God is its author, and something more than assertion is needed, to prove that HE has connected any one truth with consequences necessarily hurtful to his creatures. IF IT IS FALSE, its consequences may and *must be* bad; but then the way to get rid of them is to *prove it false*, in which case, the consequences will fall along with it into one common grave, and give trouble to no one. We may add, however, that to our minds it seems to leave materialism and fatalism precisely where it found them, and to plant religion on the imperishable basis of adaptation to the constitution which God has given to the mind of man.

## Figures and Tables

**Figure f1:**
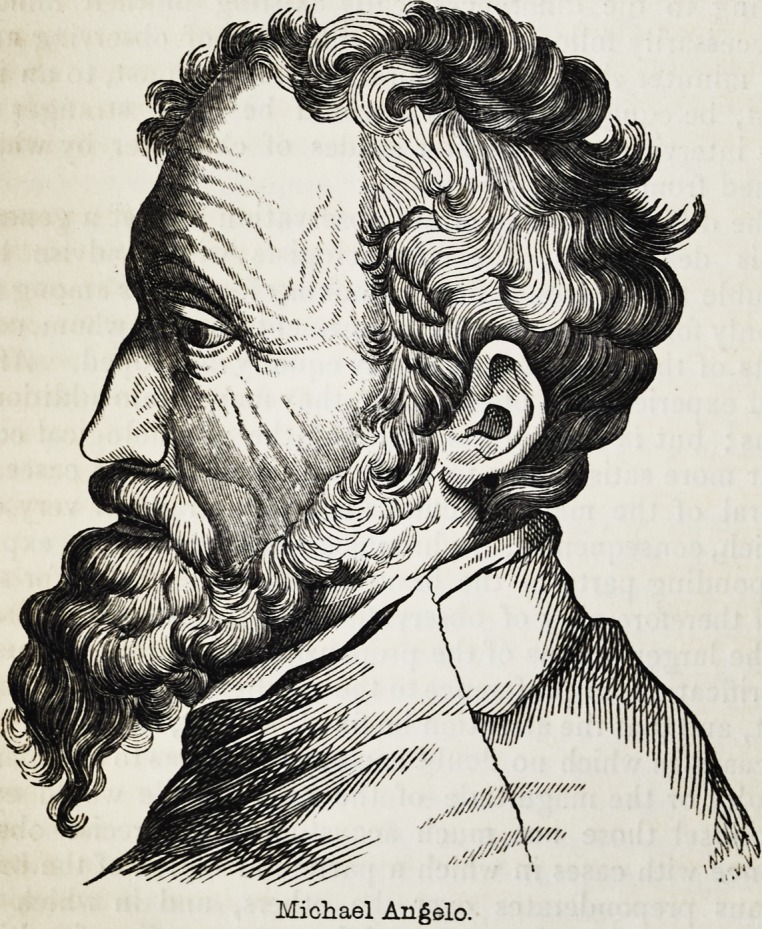


**Figure f2:**
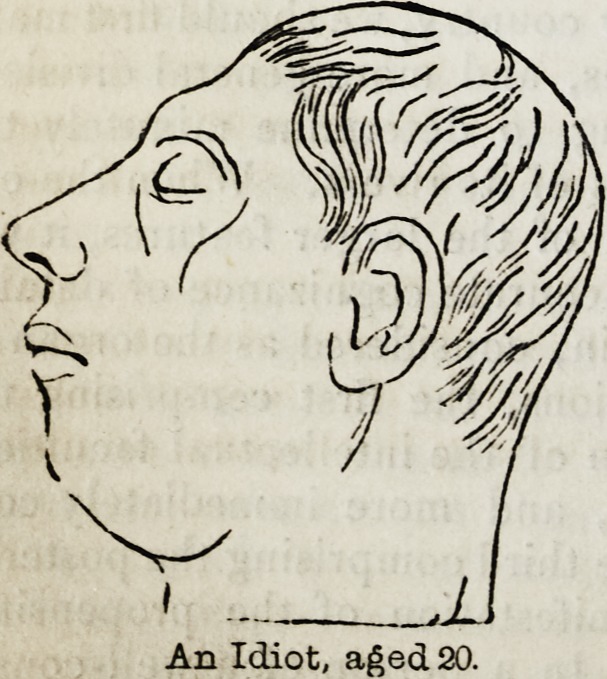


**Figure f3:**
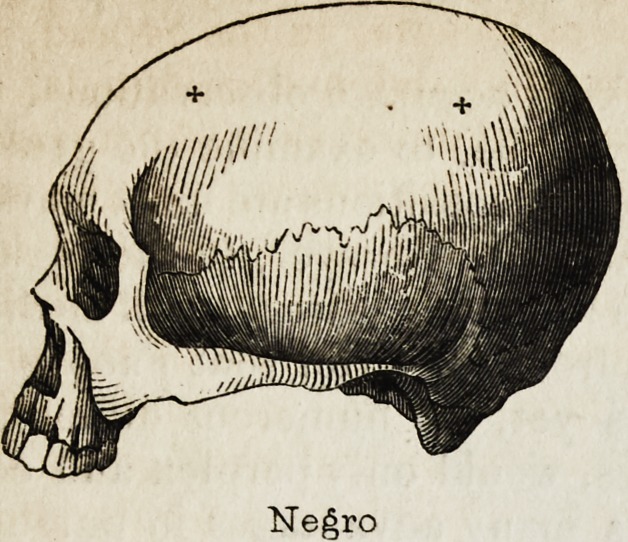


**Figure f4:**
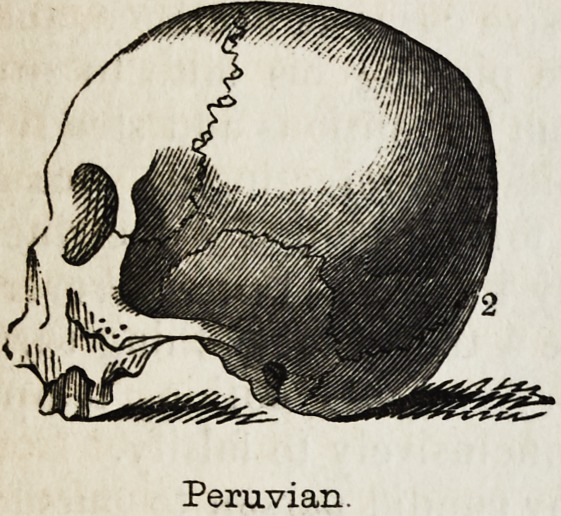


**Figure f5:**
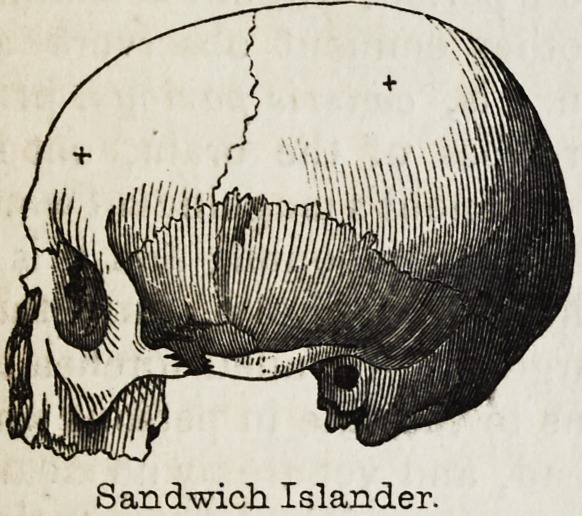


**Figure f6:**
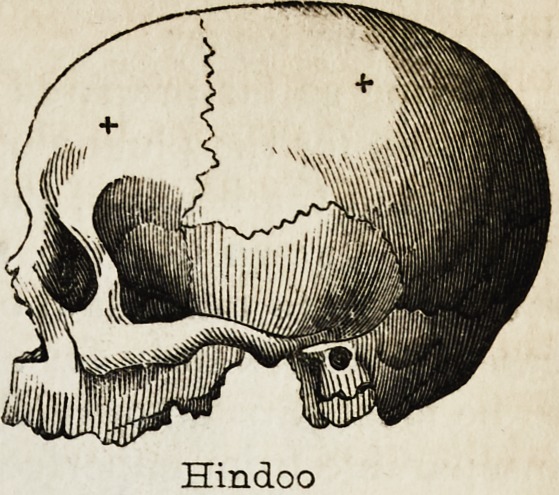


**Figure f7:**
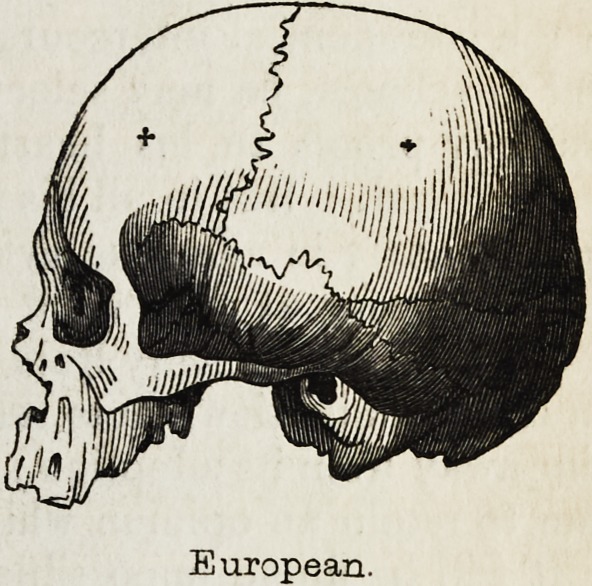


**Figure f8:**